# Attitude and personality trait changes among nursing students and professionally active nurses working with older adults

**DOI:** 10.3389/fpubh.2025.1581281

**Published:** 2025-09-15

**Authors:** Carlos Dosil-Díaz, César Bugallo-Carrera, Manuel Gandoy-Crego, Noelia Gerbaudo-González

**Affiliations:** University of Santiago de Compostela, Santiago de Compostela, Spain

**Keywords:** attitudes, personality traits, nursing students, nursing professionals, longterm care facilities for the older adult

## Abstract

We aimed to analyze if there are attitude changes toward older adults among nursing students and professionally active nurses, their attitudes regarding their job, and the personality traits based on their desire to remain in their current center. The study included 1,047 participants. The attitude toward people of an older age varied between the two study groups with differences in health- and motivation-related factors and no differences in personality-related traits attributed to older adults. Significant differences were seen in the personality traits agreeableness, awareness, and negative emotions; specifically, professionally active nurses showed higher levels of agreeableness and awareness, while negative emotions were observed more frequently among nursing students. Is possible to identify a profile that allows determining which variables are present in these professionals working with older adults that provide more satisfaction should they continue in their current job. Thus, professionally active nurses who wish to remain at their job have greater levels of engagement, a more appropriate adaptive performance, and more positive personality traits.

## Highlights

- Attitudes and personality dimensions toward adults of an older age differs between the two study groups, nursing students and professionally active nurses.
- Attitudes toward aging will be less negative in professionally active nurses that in nursing students.
- Is possible to identify a profile that allows determining which variables are present in professionally active nurses working.

## Introduction

The world’s population aging and longevity are significantly increasing in developed and developing countries (34). It is estimated that by 2050, the number of people aged > 65 years will be around 2 billion (1). Currently, 21% of the Spanish population is older than 65 years, i.e., 9 million people (1). This age shift has contributed to an increased level of professionalization in the health care of older adults over the past years (2). Nursing professionals are the cornerstone when it comes to the care in long-term facilities (3), were - among other tasks - these specialists develop direct care functions and promote healthy habits among the people they are in charge of.

Professional care improvement requires specific knowledge and skills, as well as very concrete personality traits.

A basic element for the adequate development of the work of nurses is having an emotionally positive attitude toward older adults (4). A study by Álvarez-Dardet et al. (5) shows that the level of education is a particularly relevant aspect when explaining negative concepts in young people and adults on older adults. Thus, individuals with a high level of education have less degree of old age-related stereotypes.

Seresht et al. (6) reported that first year nursing students have negative attitudes toward older adults, while more advanced students show neutral attitudes. Contrarily, licensed professionally active nurses showed positive attitudes toward the elderly. Eltantawy (7) communicated very similar observations. The author found a statistically significant relationship between students’ knowledge on aging, attitude toward people of an older age, and their will and intent to work and care of the elderly; moreover, the author described differences between the positive attitudes of first and last year nursing students, with the latter having more positive attitudes.

Contrarily, Amah and Oyetunde (8) found that nurses have negative attitudes when it comes to the care of older adults despite having a relatively good knowledge on geriatric care. Among other causes, they attribute this disagreeable attitude to the lack of personnel - which makes the care of the elderly more difficult - and the frequent behavior changes in older adults.

Alsenany (9) observed a favorable positive attitudes of Saudi nursing students toward older adults. Their attitude seems to be greatly influenced by cultural and religious factors, besides being associated to cultural influence and level of knowledge on specific aspects regarding aging. Sarabia Cobo and Castanedo Pfeiffer (10) investigated changes of negative old age-related stereotypes and myths in third year nursing students before and after taking the course *Nursing in Aging*. The authors found significant changes of these stereotypes.

Similarly, Miller and Reid (11) studied the influence of training and experience on the quality of care provided to institutionalized older adults. The research shows that nursing students, being still in training, are less able to manage negative emotions and emotional challenges related to caring for older adults compared to experienced nurses, who have developed a greater sense of empathy and emotional control.

Mellor et al. (12) communicated that although in Australia nurses who work in Multipurpose Health Services have very positive attitudes toward older adults, they lack sufficient knowledge on key clinical areas regarding nursing in geriatrics and of understanding the socio-economic context of the aged population.

Celis et al. (30) indicate that there is no clear agreement in the outcomes of the different available studies on attitudes toward older adults, further studies are needed. Despite the importance of professional nurse’s attitudes to perform adequately his/her job, and the existing evidence regarding negative attitudes toward the elderly, the topic has not been studied in-depth.

Another important aspect is to know the personality traits that define nursing students and professionally active nurses working with older adults. Yazdanian et al. (13) analyzed the personality traits of nurses and their attitudes toward people of an older age. The authors found that the variables agreeableness and neuroticism of the Big Five Personality test had a significant positive association with the attitude of the nurses toward the elderly. Similar results were reported by Valenzuela Quevedo and Yañez Salcedo (14); moreover, they found no statistically significant correlations between the personality traits responsibility, openness, and extroversion of the nurses in an emergency department with their attitude toward the care of older adults.

In a large review study, Kennedy et al. (15) conclude there is evidence that personality differences between nurses associate with their nursing specialty election, the level of job satisfaction, and their level of stress and fatigue. Considering the variety of nursing specialties, roles, context of the practice, etc., further research is needed.

Ferreira and Martino (16) hold that nursing students, as well as all other groups of nurses who are professionally active, lack education training. Doherty-King and Bowers (17) supported this. They reported that over the years the medical care provided to older adults has been severely threatened by stereotyped negative attitudes and nurses´ misconceptions. Consequently, older patients may develop a negative view toward nurses and nursing services in medical care contexts.

Thus, good preparation is key for nursing students to perform their duties successfully in the future. Several studies (7, 18, 31, 32) show that the attitudes and knowledge nurses have on aging may affect their expectations regarding their work life and the way the care for and approach older adults.

## Hypothesis and aim of the study

Our research focused on the possible changes in attitude toward people of an older age among nursing students and professionally active nurses, and how these attitudes in relation to old age, their working position, and the personality traits of the workers in the centers for elderly persons affect their wish to continue working in the center. Hypotheses:

*Hypothesis 1*: Attitudes toward aging will be less negative in professionally active nurses than in nursing students. We hypothesize that contact with people of an older age will promote positive attitudes toward this population and the same will occur with personality variables. Professionally active nurses will obtain higher scores than students in factors that imply positive treatment to the senior, specifically with the personality traits responsibility, openness, and extroversion, as well as in their attitude toward the care of older adults.

*Hypothesis 2*: In professionally active nurses working with older adults who highly desire to continue with their job, greater scores will be obtained in the personality traits agreeableness, positive emotionality, and open-mindedness, in attitudes toward the aged, and in several characteristics linked with organizational behavior (e.g., adaptive performance and engagement) in comparison to individuals who do not want to continue with their current work.

*Hypothesis 3*: It is possible to identify a profile to determine which variables characterize nurses working with older adults that imply greater satisfaction to continue in their current work.

## Methods

### Participants

One thousand forty-seven people participated in this study; 225 were male (*N* = 225. 21.5%) and the rest were women (78.5%). Mean age was 30.12 years (range 17–73 years, SD = 13.29). Around half of the participants (*N* = 534; 51%) were nursing students; 49% were professionally active nurses. An informed consent was drawn up, and all participants agreed to participate.

### Instruments

The following instruments were applied:

- A Questionnaire for the collection of personal and work data *ad hoc*.
- The Questionnaire on Negative Stereotypes about Old Age (*Cuestionario de Estereotipo Negativos hacia la Vejez - CENVE*) developed (19). The authors reported internal consistency of the factors. For this study, after collecting the data we calculated Cronbach’s alpha indexes. Results were as follows: global scale, 0.88, and 0.84, 0.81, and 0.84 for each factor. This questionnaire allows obtaining a global score (15 items) and one for each of its three five-item sub-scales: health, social motivation, and character-personality. The answers were collected using a Likert-type scale with four possible alternatives, from 1 (strongly disagree) to 4 (strongly agree). An example of an item (the first in the health sub-scale) is: “*Once they reach an age of around 65 years, most people begin to show significant memory decline*.”
- The Big Five Inventory-2- Short test BFI-2-S (20). It is the abbreviated version of the original questionnaire developed by Caprara et al. (21) who identified five personality traits: extraversion, agreeableness, conscientiousness, emotional stability (also known inversely as *neuroticism*), and openness to experience. We used the abbreviated version that comprises 30 items. Multiple studies confirm its reliability and validity. The collected data were used to determine Cronbach’s alpha indexes: extraversion, 0.79; agreeableness, 0.77; conscientiousness: 0.85; negative emotionality: 0.80; and open-mindedness, 0.80. Answers were collected using a Likert-type scale with five possible alternatives, from 1 (strongly disagree) to 4 (strongly agree). An example of an item (number 1 of extroversion) is: “*I tend to be quiet*.”
- The Adaptive Performance Scale: We used the Spanish adaptation of Ramos-Villagrasa et al. (22). This instrument has eight items and describes positive behaviors that may have been used at work. The answers were collected using a Likert-type scale with six possible alternatives, from 1 (have never done it), 2 (completely ineffective) to 6 (completely effective). Cronbach’s alpha index was 0.87.
- The Utrecht Work Engagement Scale. We used the validated Spanish version by Schaufeli and Bakker (23). This scale is composed of 17 items and has three subscales: vigor (six items), dedication (five items), and absorption (six items). The participants were asked to answer if they had felt the way expressed in the statement. If the person has never felt as stated, he/she had to answer “0” (zero) and if otherwise he/she had to indicate how many times (maximum number 5). Thus, it is a Likert-type scale with six possible alternatives; e.g., for item number 1: “*At my work I feel bursting with energy*.” Reliability, calculated using Cronbach’s alpha index, was 0.89.
- Intention to remain employed in the center. Lastly, we tested to what point the participant wished to remain in his/her workplace. For this, we created one item: “*If the decision depended on you, would you continue to work in this center?*” The answers were collected using a Likert-type scale with 11 possible alternatives; from 0 to 10, with 0 being “I want to leave immediately” and 10 “I would like to stay as long as possible.” Being a single-item measure, it was not possible to estimate the reliability.

### Procedure

We created a virtual questionnaire for nursing students and professionally active nurses. The search of professionally active nurses was done with the help of a firm that manages centers for older adults; the nursing students were found at University in Spain; all participants lived in Galicia (Spain). Once a potential participant was identified, he/she was sent a link (Microsoft platform) where (a) the aim of the study was presented, (b) the potential participant was notified that the answers would be treated anonymously, and (c) was asked to give his/her consent to participate in the study. If the subject declined to take part in the research, the survey was ended. All data were exported to SPSS version 26. Descriptive, reliability, and logistic regression analyses were performed (results were transformable to ANOVA).

### Declaration of interest statement

Each study participant read a brief description of the study and gave his/her informed consent. The study was approved by the Research Center Ethics Committee University (Ethics Committee of the University of Santiago de Compostela with approval number: GA1345436). Prior to performing the investigation, permission was requested from the Research Center Ethics Committee and was granted by the corresponding research Center of the university. The research meets the ethical principles of the Declaration of Helsinki and the American Psychological Association.

The signing authors have complied with ethical requirements and copyright clearance of the manuscript, with the research data being collected during the months of February, March, April, and May 2023.

## Results

Study participants were either nursing students or professionally active nurses working with adults of older age. Table 1 summarizes the descriptive statistics [median and standard deviation (SD)] of the study variables for both groups.

**Table 1:** Attitudes toward aging and personality traits in nursing students and professionally active nurses.

		*CENVE*	*CENVE* I	*CENVE* II	*CENVE* III	Extroversion	Agreeableness	Awareness	Emotionality negative	Openness
Nursing students	M	34.59	11.71	11.26	11.61	20.94	23.85	21.49	18.42	20.96
	SD	7.53	2.81	2.89	3.08	4.44	3.82	4.46	4.49	4.43
Professionally active nurses	M	32.39	10.62	10.52	11.23	21.23	24.43	23.07	16.59	20.61
	SD	8.79	3.33	3.14	3.35	4.09	3.52	4.58	4.23	4.14
Global	M	33.51	11.17	10.90	11.42	21.08	24.14	22.27	17.52	20.79
	SD	8.24	3.12	3.04	3.22	4.27	3.68	4.59	4.46	4.29

M, Mean; SD, Standard Deviation; CENVE, Questionnaire on Negative Stereotypes about Old Age (Cuestionario de Estereotipo Negativos hacia la Vejez).

Attitudes toward older adults were more positive in nursing students (regardless if the differences were significant or not, which will be further analyzed later) than in professionally active nurses (Table 1). This was observed globally, as well as in each of the three factors that make up the *CENVE* (health, social motivation, and personality). Regarding the five personality variables, higher levels of extroversion, agreeableness, and were found in professionally active nurses, while higher levels of negative emotionality and mental openness were seen in nursing students.

Student’s *t*-test was used to examine how significant the differences in scores were between both groups (Table 2).

**Table 2:** Score differences between nursing students and professionally active nurses (Student’s *t*-test).

	*t*	df	Sig. (bilateral)	Mean difference	Typical error difference
*CENVE*	4.34	1,007.47	0.00	2.20	0.50
*CENVE* I (Health)	5.64	1,002.49	0.00	1.07	0.19
*CENVE* II (Vigor)	3.96	1,030.14	0.00	0.74	0.19
*CENVE* III (Personality)	1.91	1,045	0.06	0.38	0.20
Extraversion	−1.07	1,043.16	0.28	−0.28	0.26
Agreeableness	−2.51	1,045	0.01	−0.57	0.23
Awareness	−5.66	1,045	0.00	−1.58	0.28
Emotionality negative	6.77	1,045	0.00	1.82	0.27
Openness	1.30	1,045	0.19	0.34	0.26

df, degrees of freedom; sig., significance.

Significant overall differences in the attitudes toward older adults between the two groups were found, as well as for the factors health and vigor. The opposite was observed for personality. I.e., nursing students were more positive in their attitudes toward people of an older age in comparison to professionally active nurses. Regarding personality variables, significant differences were seen for agreeableness, conscientiousness, and negative emotionality; professionally active nurses have higher levels of agreeableness and conscientiousness, while nursing students obtained higher scores for negative emotionality.

We next carried out a factor analysis based on the intention to remain employed at the center; for this analysis, only professionally active nurses were considered. The principal component method was used as the factor analysis technique and the rotation method based on Varimax. The number of factors to identify was not fixed, but data had to meet two criteria: have a self-value > 1 and an explained variance > 0.05.

Table 3 shows the information on variable saturation for each significant factor for the total sample of professionally active nurses. We identified three factors that explain 27.10, 18.35, and 13.26% of total variance (i.e., 58.71%). The first factor clusters all engagement variables and the variable awareness. The second factor, all negative attitude factors toward older adults, that is, the scale and sub-scales of the *CENVE*. The third group clusters extroversion, negative emotion with a negative sign (i.e., emotionality positive), open-mindedness, and adaptive performance. Thus, the three may be identified as (1) engagement; (2) negative attitudes toward aging; and (3) adaptive performance and appropriate personality traits for social interaction.

**Table 3:** Factor analysis of professionally active nurse (Varimax rotation method).

	Component		
	1	2	3
*CENVE* I (Health)	0.01	0.86	−0.19
*CENVE* II (Social motivation)	0.14	0.85	−0.03
*CENVE* III (Personality)	−0.01	0.88	0.08
Extraversion	0.07	0.09	0.83
Agreeableness	−0.03	−0.13	−0.03
Awareness	0.38	0.22	0.20
Emotionality negative	0.12	−0.08	−0.44
Openness	0.05	−0.15	0.48
Engagement UWES	0.99	0.03	0.07
Engagement vigor	0.87	0.03	0.07
Engagement dedication	0.86	0.07	0.08
Engagement absorption	0.89	0.00	0.04
Adaptive performance APS	0.16	−0.07	0.69

We compared the factor analysis scores (Student’s *t*-test) between students with high and low desire to continue working at the center. Statistically significant differences were identified between both groups in the factor *engagement* (Factor 1) (Table 4); that is, professionally active nurses who express their intention to remain at their current work have higher levels of engagement. However, on the attitudes toward aging, no significant inter-groups differences were found. Regarding the factor adaptive performance and social interaction appropriateness, significant inter-group differences were seen. Professionally active nurses who are more willing to continue working in their current center had more positive scores in the Big Five Personality test personality dimensions linked with interactions with others, as well as a more adequate level of adaptive performance.

**Table 4:** Comparisons between professionally active nurses with high or low desire to continue working at their current center (Student’s *t*-test).

	Student’s *t*-test				
	*t*	df	Sig. (bilateral)	Mean difference	Typical error difference
Factor 1: engagement	−9.15	330	0.00	−0.87	0.09
Factor 2: negative attitudes towards aging	0.22	330	0.82	0.02	0.11
Factor 3: adaptive performance and social interaction appropriateness	−2.03	330	0.04	−0.22	0.11

df, degrees of freedom; sig., significance.

## Discussion

The outcomes of this study allow us to conclude the following:

Attitudes toward adults of an older age differs between the two study groups – nursing students and professionally active nurses-, which is in line with the results communicated by Seresht et al. (6). It is an overall trend, as well as for two out of the three factors included in the scale used to examine attitudes toward older adults. Specifically, the differences occur for the factors health and motivation, but not for personality traits attributed to older adults. These results support the first part of Hypothesis 1 (attitudes toward aging will be less negative in professionally active nurses that in nursing students) and are in line with the studies by Oyetunde et al. (18).

Hypothesis 1 stated is supported by the research of Miller and Reid (11) where they studied the influence of training and experience on the quality of care provided to institutionalized older adults. The research shows that nursing students, being still in training, are less able to manage negative emotions and emotional challenges related to caring for older adults compared to experienced nurses, who have developed a greater sense of empathy and emotional control.

Research by Egilegor et al. (24) also supports Hypothesis 1. This study concludes that nursing students often lack knowledge about older adults and need opportunities to develop positive attitudes toward them, compared with nursing professionals, who believe that the more contact they have, the lower their negative attitudes toward older adults and aging. In the present study, nursing professionals obtained a higher mean; these results may be due to their greater professional experience in caring for older adults.

Concerning personality variables, there are significant differences between nursing students and professionally active nurses in agreeableness, awareness, and negative emotions. Thus, partly, the second part of Hypothesis 1 is confirmed.

Professionally active nurses have higher agreeableness and awareness levels. This is in line with the results by Yazdanian et al. (13), who observed that the variable *kindness* significantly and positively associates with the attitude of the nurses toward older adults, although this was not the case for negative emotions, for which nursing students had higher values. These results are supported by Tipacti (25), who found that students’ attitudes toward old age were mostly negative. This coincides more specifically with a study conducted by Hernández et al. (26) and Zenhari et al. (33).

However, these results are not in agreements with those in a study by Valenzuela Quevedo and Yañez Salcedo (14), who hold they found similar results between professionally active nurses and nursing students. Moreover, they found no statistically significant correlation between the personality traits *responsibility*, *openness*, and *extroversion* in professionally active nurses working in the emergency department and their attitude toward the care of older adults. These results are supported by Mojarrad and Mehrabian (27), who explain that personality traits such as kindness and conscientiousness affect the quality of care provided to older patients. Practicing nurses tend to display higher levels of empathy and professionalism, while nursing students may lack this skill due to their lack of experience dealing with institutionalized patients. These results are consistent with those of Miller and Reid (11) demonstrate that nursing students are less able to manage negative emotions and emotional challenges related to caring for the elderly compared to experienced nurses.

Kennedy et al. (15) demonstrate that these differences in personality traits associate with the selection of the nursing specialty, the level of job satisfaction, and stress.

In the comparison of professionally active nurses with high or low desire to continue working in the center, we conclude that it is possible to identify a profile that allows determining which variables are present in professionally active nurses working with older adults that provide more satisfaction as to want to continue in their current job.

There are three relevant factors: commitment, negative attitudes toward aging, and adaptive performance and adequate social interaction personality traits. This supports Hypothesis 2.

Our results also support hypothesis 3, except for attitudes toward aging, which are negative with no inter-group differences. However, professionally active nurses who wish to continue with their current work have higher levels of commitment, a more adequate adaptive performance, and personality traits values that are more positive. These outcomes are partly in line with those described by Mellor et al. (12), who communicated that professionally active nurses have very positive attitudes toward older adults, but have clinical geriatric nursing knowledge deficits and of understanding the socio-economic context of the aged population.

Other research support hypothesis 3, such as that of Lu et al. (28) determines that it is possible to identify a profile of nurses who work with older adults and who present greater job satisfaction, as well as the studies of Bishop et al. (29), which detail the different factors that affect nurses’ job satisfaction and determine their professional development.

There are several limitations to this study:

- Although the sample size of nursing students was adequate (~50% of the total sample), we were unable to assess their organizational behavior. We show there are no inter-group differences regarding attitudes toward older adults and thus, further studies should focus only on professionally active nurses.
- Organizational behavior can be measured using a broad set of variables. We show that commitment and adaptive behavior are key for these type of studies; however, it is impossible to rule out that in the future other variables may be provide additional information regarding professional performance.
- We show that both study groups have negative attitudes toward aging; thus, further studies should provide solutions to prevent estereotyping.

## Conclusion

The results of this research show significant differences between nursing students and practicing nurses in how they manage kindness, awareness, and negative emotions toward institutionalized older patients. Experienced nurses have developed more advanced emotional skills and therefore have a more positive and competent attitude toward older patients. Students, on the other hand, are still in training and often face greater emotional difficulties when interacting with this vulnerable group, which may be reflected in a higher incidence of negative emotions and lower empathy.

The advantage of attitude-, personality-, and organizational behavior-related variables is that they allow selecting better the nursing staff when the aim is to remain in the job, a matter of great relevance for any center. People who are more willing to stay at their job may be identified through personality tests and by determining their level of commitment and adjusting to the work position. This study may be of interest for people who make use of nursing services and for the profession in general.

## Data Availability

The raw data supporting the conclusions of this article will be made available by the authors, without undue reservation.
